# Coloration of the Chilean Bellflower, *Nolana paradoxa*, interpreted with a scattering and absorbing layer stack model

**DOI:** 10.1007/s00425-015-2395-0

**Published:** 2015-09-14

**Authors:** Doekele G. Stavenga, Casper J. van der Kooi

**Affiliations:** Computational Physics, Zernike Institute for Advanced Materials, University of Groningen, Nijenborgh 4, NL-9747 AG Groningen, The Netherlands; Plant Physiology, Groningen Institute for Evolutionary Life Sciences, University of Groningen, Nijenborgh 7, NL-9747 AG Groningen, The Netherlands

**Keywords:** Pigments, Integrating sphere, Kubelka–Munk theory, Transmittance and reflectance spectra, Pollination

## Abstract

**An absorbing-layer-stack model allows quantitative analysis of the light flux in flowers and the resulting reflectance spectra. It provides insight in how plants can optimize their flower coloration for attracting pollinators.**

The coloration of flowers is due to the combined effect of pigments and light-scattering structures. To interpret flower coloration, we applied an optical model that considers a flower as a stack of layers, where each layer can be treated with the Kubelka–Munk theory for diffusely scattering and absorbing media. We applied our model to the flowers of the Chilean Bellflower, *Nolana paradoxa*, which have distinctly different-colored adaxial and abaxial sides. We found that the flowers have a pigmented, strongly scattering upper layer, in combination with an unpigmented, moderately reflecting lower layer. The model allowed quantitative interpretation of the reflectance and transmittance spectra measured with an integrating sphere. The absorbance spectrum of the pigment measured with a microspectrophotometer confirmed the spectrum derived by modeling. We discuss how different pigment localizations yield different reflectance spectra. The absorbing layer stack model aids in understanding the various constraints and options for plants to tune their coloration.

## Introduction

Floral coloration is in virtually all plant species due to the expressed pigments that selectively absorb incident light in a restricted wavelength range (e.g. Kay et al. 1981; Lee 2007). For example, flowers with the blue-absorbing carotenoids are yellow and flowers with blue-green-absorbing anthocyanins are purple [reviewed by Grotewold (2006)]. Due to the irregular shape and ordering of the petal’s components (e.g. vacuoles) incident light is backscattered in many directions. The diffuse scattering thereby provides a display that is similarly visible under many angles (Wehner and Bernard 1993; Lee 2007; van der Kooi et al. 2015a).

The pigments together with the structure of the cell complexes inside the petals and the flower’s thickness determine the proportion of light reflected by the flowers. Additionally, the epidermal surface structure slightly contributes to the flower’s appearance. When the surface is smooth and flat the flower is glossy, and with a papillose surface the flower is matte (van der Kooi et al. 2014; Papiorek et al. 2014). In other words, the visual signal of flowers depends on the spectral properties and concentration of the expressed pigments, but also on the structure of the inner elements and surface.

Many studies have focused on flower coloration because of its importance for visual signaling to pollinators. The overall floral reflectance has been documented for numerous plant species (e.g. Arnold et al. 2010; Dyer et al. 2012), but rather few quantitative treatments have been attempted to advance our understanding of how light interacts with the inner components of flowers (Lee 2007). Exner and Exner (1910) applied geometrical optics to various flower structures in a first attempt to explain flower coloration (see also Kay et al. 1981; Gorton and Vogelmann 1996; Lee 2009; Gkikas et al. 2015). However, the inhomogeneity of the flower interior makes a detailed optical analysis cumbersome. The presently available powerful computational methods in principle allow calculation of the spectral signatures of any complex structure, but the required knowledge of the spatial distribution of the essential optical parameters, that is, the refractive index and absorption coefficient, is unavailable for any flower. The contribution of the different structures and layers of flowers to the overall visual signal is therefore currently unknown.

Studies on plant leaves showed that the Kubelka–Munk theory for absorbing and diffusely scattering media offers an alternative approach (Kubelka and Munk 1931; Allen et al. 1969a, b). The central parameters in the Kubelka–Munk theory, the absorption and scattering coefficients of the leaves, could be derived from measured reflectance and transmittance spectra (Allen et al. 1969a, b). Yamada and Fujimura (1991) expanded this method by treating a leaf as a stack of plates, where each plate has its individual optical properties, which allowed to non-invasively estimate the chlorophyll content of dicotyledonous leaves.

Here, we present a related spectral analysis in which we treat flowers as a stack of absorbing and scattering optical plates. As a basic and exemplary species for our approach we chose the Chilean Bellflower, *Nolana paradoxa* Lindl., because the flower’s anatomy can be considered as consisting of only two, yet quite differently structured layers. Notably the purplish pigment in *N. paradoxa* occurs only in the upper epidermis, causing a distinctly different coloration of the adaxial and abaxial sides. With the combined Kubelka–Munk-layer-stack method we could straightforwardly assess the spectral absorbance of the flower’s pigment. We discuss how the model allows a quantitative analysis of the virtues of various coloration strategies of flowers.

## Materials and methods

### Flowers and photography

**Fig. 1:**
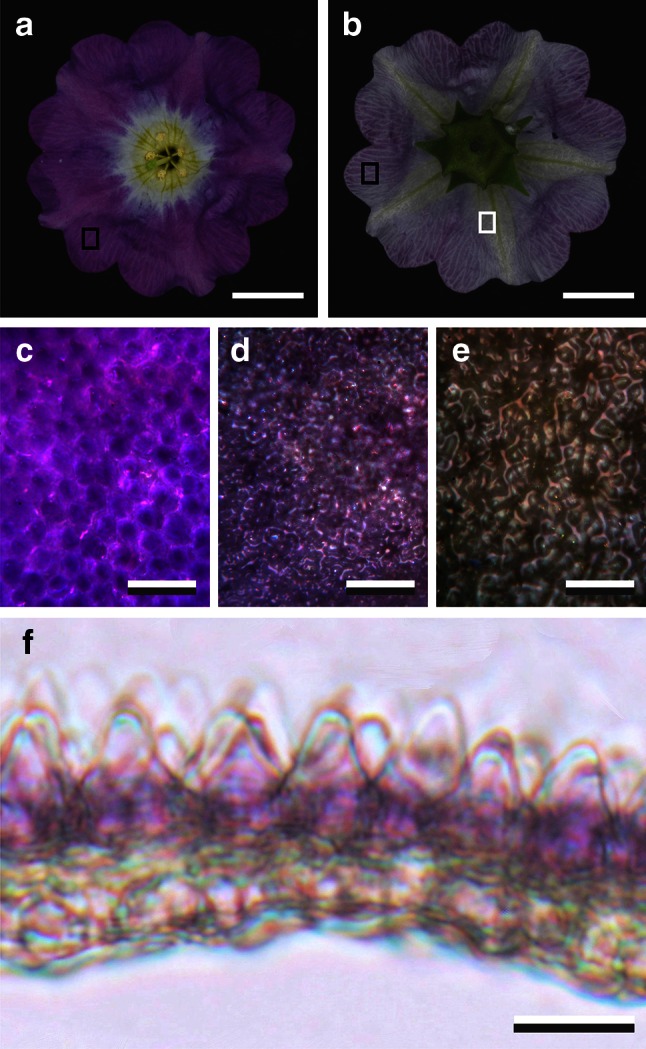
Flower of the Chilean Bellflower *Nolana paradoxa* and corolla pigmentation. **a** Photograph of the adaxial side of the flower. **b** Photograph of the abaxial side of the flower. **c** Epi-illumination micrograph of the adaxial epidermis, indicated by the black rectangle in (**a**). **d**, **e** Epi-illumination micrographs of the abaxial epidermis in the violet (*black rectangle* in **b**) and white area (*white rectangle* in **b**). **f** Cross section of the corolla. *Scale bars* **a**, **b** 1 cm; **c**–**f** 50 µm

*Nolana paradoxa* plants were grown from seeds in pots with ample irrigation and temperature 20 ± 2 ºC under a light regime of L:D = 16:8 in the greenhouse of the Department of Plant Physiology, Groningen, Netherlands. They were photographed with a Nikon D70 digital camera equipped with an F Micro-Nikkor (60 mm, f2.8; Nikon, Tokyo, Japan) macro objective (Fig. 1a, b). Details of the flower’s epidermal surfaces were photographed with a Zeiss Universal Microscope (Zeiss, Oberkochen, Germany) using an Olympus20/0.45 objective (Olympus, Tokyo, Japan) (Fig. 1c–e).

### Floral anatomy

To examine the structure as well as the pigment distribution, we examined cross sections of the corolla of *N. paradoxa*. Small pieces of the flower were therefore embedded in a 6 % solution of agarose (Duchefa, Haarlem, Netherlands) at ~55 °C, i.e. near the solidification temperature, to provide mechanical support to the small floral elements (following Zelko et al. 2012). Together with the agarose, the floral element was cut to ~200 µm thick sections using a sharp razor blade. The sections were then photographed with the Zeiss Universal Microscope in transmission mode (Fig. 1f).

### Spectrophotometry

To measure the reflectance and transmittance of the flower corolla, we used an integrating sphere. A deuterium-halogen lamp (Avantes AvaLight-D(H)-S) delivered light, via an optical fiber, to the integrating sphere (AvaSphere-50-Refl). A corolla piece, positioned at the aperture of the integrating sphere, was directionally (and about normally) illuminated from within the sphere at an area with diameter ~5 mm. The reflected light was collected by a second optical fiber, connected to an Avaspec-2048 CCD detector array spectrometer (Avantes, Eerbeek, Netherlands). A white diffuse tile (Avantes WS-2) was used as a reference. For transmittance measurements, an area of about 1 mm of the same corolla piece was illuminated from outside the sphere via an optical fiber. The absorbance spectrum of the flower’s pigment was measured on corolla pieces immersed in water with a microspectrophotometer (MSP), consisting of a xenon light source, a Leitz Ortholux (Leitz, Wetzlar, Germany) microscope and the spectrometer mentioned above. The microscope objective was an Olympus 20/0.45. The measurement area was typically a square with side length ~10 µm. Due to the MSP optics, the absorbance spectra were limited to above ~350 nm.

### Flowers treated as a stack of scattering and absorbing layers

In many flowers the composing material is distributed inhomogeneously in differently structured layers, i.e., the upper epidermis, mesophyll and lower epidermis; notably the expression of pigments is often confined to specific layers (e.g. Kay et al. 1981; Lee 2007). Here we consider a flower as to consist of separate layers that can be treated with the Kubelka–Munk theory for scattering and absorbing materials (Kubelka and Munk 1931). We furthermore assumed that the optical properties of the different layers combined (i.e. the whole flower) can be treated with the theory for a stack of reflecting and transmitting plates where for each layer (with number *i*) the reflectance (*r*_*i*_) and transmittance (*t*_*i*_) are known, thus allowing calculation of the stack reflectance (*R*_s_) and transmittance (*T*_s_) (Yamada and Fujimura 1991; Stavenga et al. 2006). Because the flower’s upper and lower epidermal surfaces slightly affect the reflectance and transmittance of the flowers, they were incorporated in the combined Kubelka–Munk-layer-stack modeling. Using measured reflectance and transmittance spectra, the modeling yielded quantitative estimates of the absorption parameters ($K_{i}^{*} = K_{i} d_{i}$) and scattering parameters ($S_{i}^{*} = S_{i} d_{i}$) of the layers, and with known thicknesses (*d*_*i*_) the absorption coefficients (*K*_*i*_) and scattering coefficients (*S*_*i*_) were derived. The detailed modeling procedures are explained in the “Appendix”.

## Results

### Appearance and anatomy of *Nolana paradoxa *flowers

The flowers of the Chilean Bellflower, *N. paradoxa*, show a remarkably dissimilar coloration of the adaxial and abaxial sides. At the adaxial side, the corolla has an approximately homogeneous intense violet color (Fig. 1a), although in close-up view the violet coloration is patterned (Fig. 1c). The corolla tube has a paler color (Fig. 1a). In contrast, the abaxial side has a rather unsaturated violet color (Fig. 1b, d), with the vein areas being even more colorless (Fig. 1b, e).

To uncover the origin of the strongly asymmetric coloration, we have studied the anatomy of the corolla. Cross sections revealed that the violet pigment is mostly concentrated in the upper epidermal cells (Fig. 1f). The cells of the upper epidermis have a papillose shape, whereas the cells of the lower epidermis create a smoother profile (Fig. 1f).

### Floral transmittance and reflectance

**Fig. 2:**
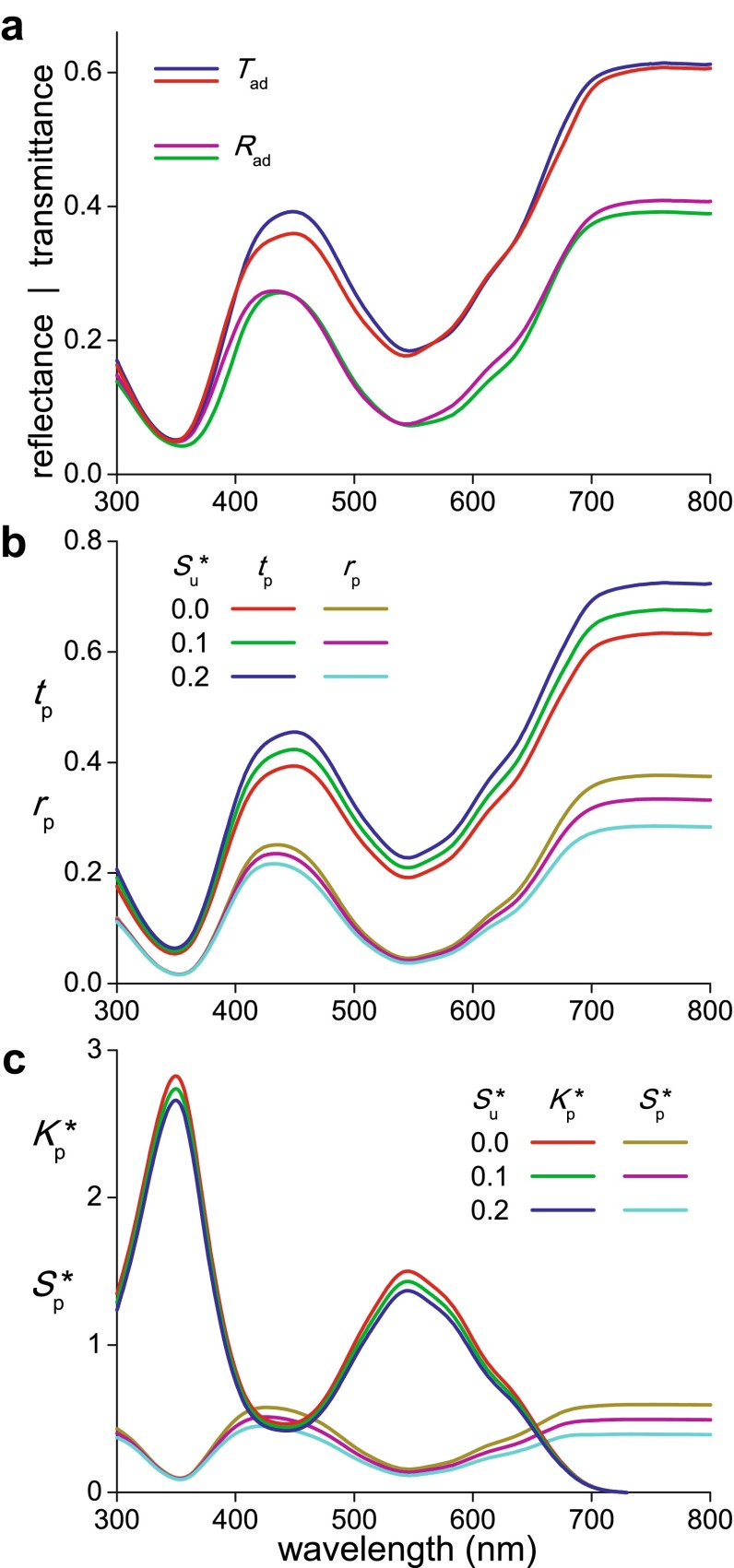
Optical characteristics of a *Nolana paradoxa* flower. **a** Two transmittance (*T*_ad_) and reflectance (*R*_ad_) spectra of the adaxial side of a corolla measured with an integrating sphere. **b** Transmittance (*t*_p_) and reflectance (*r*_p_) spectra of the pigmented layer calculated from the average of the measured transmittance ($\bar{T}_{\text{ad}}$) and reflectance ($\bar{R}_{\text{ad}}$) spectra, using three values for the scattering parameter of the unpigmented layer ($S_{\text{u}}^{*}$). **c** Absorption ($K_{\text{p}}^{*}$) and scattering ($S_{\text{p}}^{*}$) parameter for the pigmented layer calculated from the layer’s transmittance and reflectance spectra of **b**

To quantify the optical properties of the *N. paradoxa* corolla, we measured transmittance and reflectance spectra with an integrating sphere, applying first illumination of the adaxial side. Different locations yielded very similar spectra (Fig. 2a).

We interpreted the measured spectra of the corolla with a combined Kubelka–Munk-layer-stack model consisting of four layers: the upper surface (layer #1), a pigmented layer (#2), an unpigmented layer (#3), and the lower surface (#4); see the “Appendix”, Fig. 6a. First we investigated the contribution of the corolla’s surfaces. The reflectance from the petal interior is minimal in the wavelength range where absorption is extreme, that is, in the ultraviolet. The remaining reflectance, which must be mainly due to the surface, was no more than a few percent, in good correspondence with the modeling that yielded as an estimate ~0.03 for the surface reflectance in the UV. This value is quite compatible for plant material with refractive index of 1.40–1.45 facing air (Gausman et al. 1974). Assuming that the surface reflectance is about wavelength independent, we used in the further modeling for both layers 1 and 4 constant reflectance values $r_{1} = r_{4} =$ 0.03 and hence transmittance values $t_{1} = t_{4} =$ 0.97.

The anatomy indicated that layer 3 was virtually pigmentless (i.e. the absorption coefficient *K*_3_ = *K*_u_ = 0). The colorless light scattering of this layer suggested a constant, wavelength-independent scattering parameter. We considered a few different scattering parameters, $$S_{3}^{*} = S_{\text{u}}^{*}$$ = 0.0, 0.1, 0.2, or, the reflectance $$r_{3} = r_{\text{u}}$$$$= S_{\text{u}}^{*} /(1 + S_{\text{u}}^{*} )$$ = 0.0, 0.09, 0.17 and the transmittance $$t_{3} = t_{\text{u}} = 1/(1 + S_{\text{u}}^{*} )$$ = 1.0, 0.91, 0.83 (“Appendix” Eqs. 10a, 10b). We note here that the value $$S_{\text{u}}^{*}$$ = 0.0 is in fact an extreme and irrealistic value, because it means that layer 3 is negligible, as with zero absorption and scattering it does not contribute to the flower optics. Consequently, the interior of the corolla then behaves as a single, homogeneously pigmented layer.

As explained in the “Appendix” (Eqs. 16), we calculated the transmittance (*t*_p_) and reflectance (*r*_p_) spectra for the pigmented layer (#2) by combining the reflectance and transmittance properties of layers 1, 3 and 4 with the averaged transmittance ($\bar{T}_{\text{ad}}$) and reflectance ($\bar{R}_{\text{ad}}$) spectra measured of the adaxial side of the corolla (Fig. 2a, b). Subsequently, using “Appendix” Eqs. 6a–6c, we derived from the *t*_p_- and *r*_p_-spectra the absorption ($K_{\text{p}}^{*}$) and scattering ($S_{\text{p}}^{*}$) parameters of the pigmented layer (Fig. 2c). The absorption parameter spectra demonstrated that the pigment responsible for the flower’s purple color strongly absorbs in the blue-green wavelength range, much less in the violet, and very strongly in the ultraviolet. The absorption parameter spectra of layer 2 calculated with scattering parameter values $S_{\text{u}}^{*}$ = 0.0, 0.1, and 0.2 only slightly differed. The calculated scattering parameter spectra of layer 2 showed distinct troughs in those wavelength ranges where absorption is severe; the spectra varied more strongly (Fig. 2c).

### Absorbance spectrum of *N. paradoxa’s* pigment

**Fig. 3:**
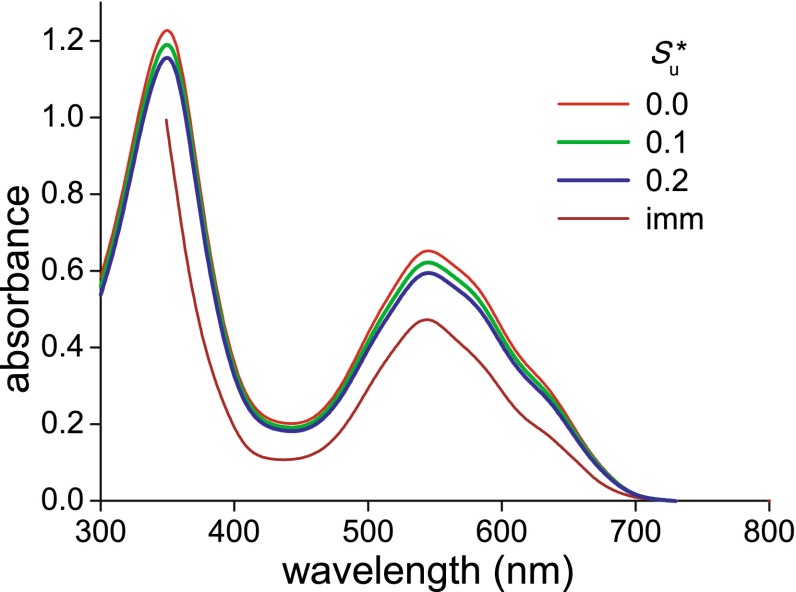
Absorbance spectrum measured by microspectrophotometry of a *N. paradoxa* corolla immersed in water (imm) compared with absorbance spectra calculated from the absorption parameter ($K_{\text{p}}^{*}$) of Fig. 2c

To investigate the pigment of *N. paradoxa* more directly, in addition to the calculations of the pigment absorption spectra using integrated sphere measurements, we immersed pieces of the corolla in water. The immersion served to remove the air from the corolla and thus, by annihilating the refractive index differences, to largely reduce light scattering by the corolla tissue. The absorbance spectrum measured with a microspectrophotometer (Fig. 3) closely resembled that of the calculated absorption parameter ($K_{\text{p}}^{*}$) spectra of Fig. 2c.

If the $K_{\text{p}}^{*}$-spectra had been measured in a homogeneously pigmented, non-scattering medium with absorption coefficient *K* and thickness *d*, the absorbance (or optical density) would have been *D* = 0.4343 *Kd* = 0.4343K* (see the “Appendix”). We therefore converted the $K_{\text{p}}^{*}$-spectra into absorbance spectra, and indeed the resulting spectra closely approximated the spectrum obtained from the immersed tissue (Fig. 3). As indicated by Fig. 3, with the latter approach we were able to determine the absorbance spectrum in the full ultraviolet–visible-wavelength range, which was not possible with our microspectrophotometer.

### Transmittance and reflectance spectra of the corolla’s abaxial side

**Fig. 4:**
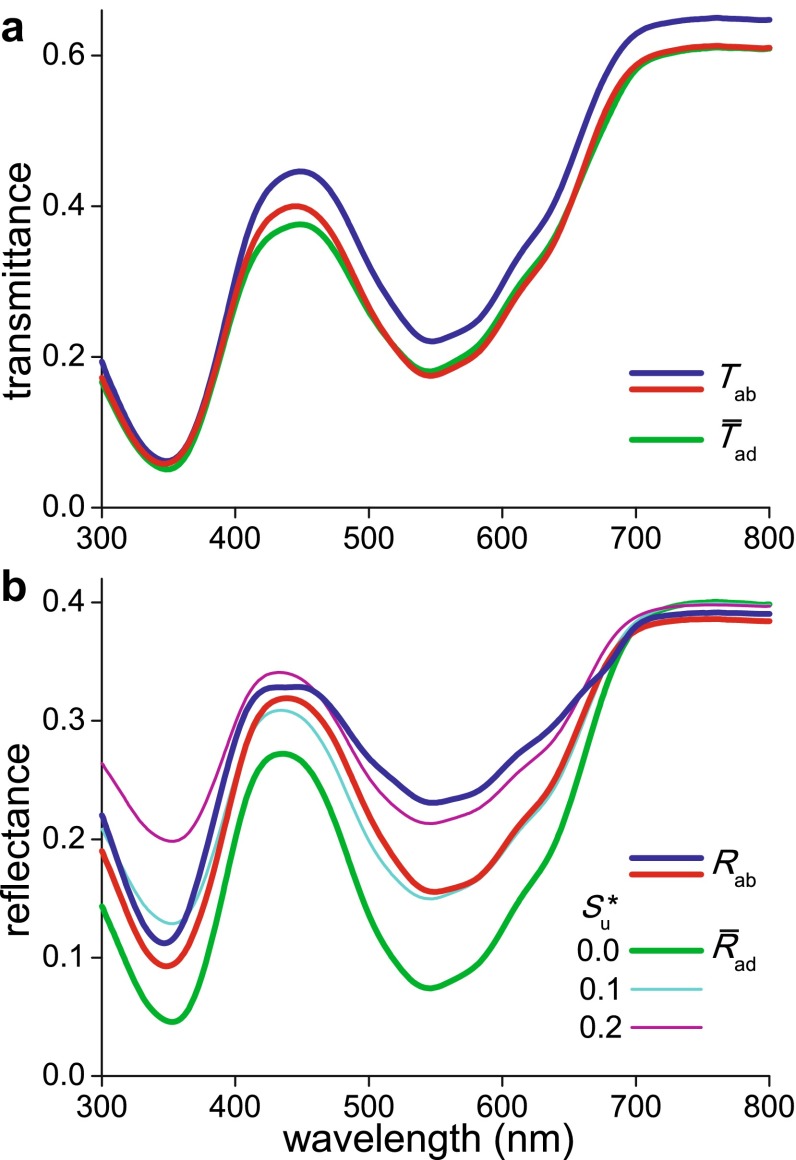
Transmittance and reflectance spectra of the abaxial side of a *N. paradoxa* corolla. **a** Two transmittance spectra measured with an integrating sphere (*T*_ab_) together with the average ($\bar{T}_{\text{ad}}$) of the two transmittance spectra measured from the adaxial side (Fig. 2a). **b** Two reflectance spectra measured with an integrating sphere (*R*_ab_; *blue* from a vein area,* red* from an area in between veins) together with reflectance spectra calculated for illumination from the abaxial side using the reflectances and transmittances of the various layers. The spectra were calculated with scattering parameter values 0.0, 0.1 and 0.2 of the unpigmented layer. The reflectance spectrum calculated with scattering parameter $S_{\text{u}}^{*}$ = 0.0 yields the averaged (measured) adaxial reflectance ($\bar{R}_{\text{ad}}$); see text

To quantitatively investigate the effect of the asymmetrical localization of the pigment on the reflectance of the opposite sides of the corolla, we also studied the case when the incident light was coming from the abaxial side. The transmittance spectra then measured with the integrating sphere (*T*_ab_) were very similar to the transmittance spectra obtained with adaxial illumination (*T*_ad_) (Figs. 2a, 4a; see also the “Appendix”). However, the measured abaxial reflectance spectra severely deviated from the adaxial spectra (Fig. 4b).

To understand the measured spectra quantitatively, we calculated the abaxial reflectance with the four layer model, using the same parameter values as in the previous modeling of the adaxial spectra. The parameter set derived with $S_{\text{u}}^{*}$ = 0.0 yielded a reflectance spectrum of the abaxial side identical to the average of the two reflectance spectra measured of the adaxial side ($\bar{R}_{\text{ad}}$; Figs. 2a, 4b); this can be directly understood, because the latter spectrum was used in the calculation procedure of the parameter value set, which implicitly assumed a homogeneous corolla. The reflectance spectrum calculated with $S_{\text{u}}^{*}$ = 0.1 corresponded well with the spectrum measured from an area in between the veins (Fig. 4b, red curve), and the spectrum calculated with a scattering parameter value $S_{\text{u}}^{*}$ = 0.2 approximated the spectrum measured from a vein area (Fig. 4b, blue curve).

## Discussion

Flowering plants advertise their presence to pollinators by displaying colorful flowers. The flower’s inner structures crucially determine the proportion of backscattered and reflected light, and the floral pigments modulate the spectral distribution of the backscattered light by wavelength-selective absorption, thereby creating spectral contrast with the surrounding plant structures (e.g. Chittka and Menzel 1992; Dyer et al. 2012; van der Kooi et al. 2015b).

To gain insight into the consequences of the various ways of pigmentation and scattering encountered in flowers, we measured reflectance and transmittance spectra of *N. paradoxa* with an integrating sphere. We analyzed the spectra with a combined Kubelka–Munk-layer-stack model by treating the corolla as a stack of four layers: two scattering layers, with pigment only in the upper layer, in between two reflecting surface layers. The modeling showed that the surface reflectance was only a few percent, which is probably due to the shape of the epidermal cells (Fig. 1f). The cone-shaped epidermal cells are generally assumed to focus incident light at their pigment (Gorton and Vogelmann 1996), thus causing the local variations in color patterns observed with epi-illumination (Fig. 1c). However, corolla pieces immersed in fluid clearly demonstrated that the pigment in the upper epidermis is variably concentrated in the epidermal cells, resulting in a somewhat patterned image in close-up view (Fig. 1c). An additional and probably functionally more important consequence of the conical shape of the epidermal cells is the scattering of incident light into a large spatial angle (van der Kooi et al. 2014; Papiorek et al. 2014), resulting in a matte display that is equally visible from many sides (Wehner and Bernard 1993; van der Kooi et al. 2015a). The local inhomogeneities of the flower interior are another important source of light scattering by the flower’s tissue.

The reflectance spectrum of the abaxial side measured at an area in between the veins could be described by a scattering parameter value of the corolla interior’s unpigmented layer $S_{\text{u}}^{*}$ ~ 0.1, in combination with a scattering parameter of the pigmented layer, which in the long-wavelength asymptote is $S_{\text{p}}^{*}$ ~ 0.5; for a vein area the corresponding values are $S_{\text{u}}^{*}$ ~ 0.2 and $S_{\text{p}}^{*}$ ~ 0.4, respectively. In other words, scattering by the pigmented layer and also by the vein areas is strong, because their interior is very inhomogeneous. Scattering by the tissue of the unpigmented layer in between the veins is relatively modest.

From the scattering parameters and the thicknesses of the two interior layers the associated scattering coefficients of the two layers follow. Taking for both layers effective thicknesses of ~40 µm (Fig. 1f), the scattering coefficients of the unpigmented and pigmented layer for the two cases become *S*_u_ ~ 2.5 and 5.0 mm^−1^ and *S*_p_ ~ 12.5 and 10.0 mm^−1^, respectively. At the green absorption peak wavelength (*λ*_max_ = 545 nm) the pigment’s absorption parameter is $K_{\text{p}}^{*}$ ~1.4, so that the absorption coefficient at the peak wavelength is ~35 mm^−1^. Interestingly, similar values were derived from other flowers (in preparation).

The modeling allowed quantitative insight into the flower tissue optics. As predicted for a layer stack, the measured transmittance spectra for different locations and side of illumination were very similar, but the reflectance spectra of both sides were very different (Fig. 4b). Reflectance spectra calculated with the parameter set derived from the adaxial measurements generally agreed with the measured spectra, but minor deviations remained. The calculations assumed that the scattering parameter in the mesophyll layer was a constant, but the scattering probably slightly depends on the wavelength. Also, the chosen approach has some limitations. Firstly, the Kubelka–Munk theory is based on the assumption that the light flux in the media is diffuse, that is, perfectly random. However, in both the reflectance and transmittance measurements with the integrating sphere, the illumination was directional and about normal to the corolla surface. The light flux in the corolla interior will become rapidly diffuse, but this might not yet be the case in the very first, epidermal layer. Furthermore, the inhomogeneously distributed pigmentation will impair a perfectly diffuse light flux. Nevertheless, the very different colors of both sides of the corolla and the associated reflectance spectra are well explained by the asymmetric pigment localization and layering of the corolla interior. An asymmetric distribution of pigments is observed frequently throughout the plant kingdom (e.g. Lee 2007) and may serve an economical function.

**Fig. 5:**
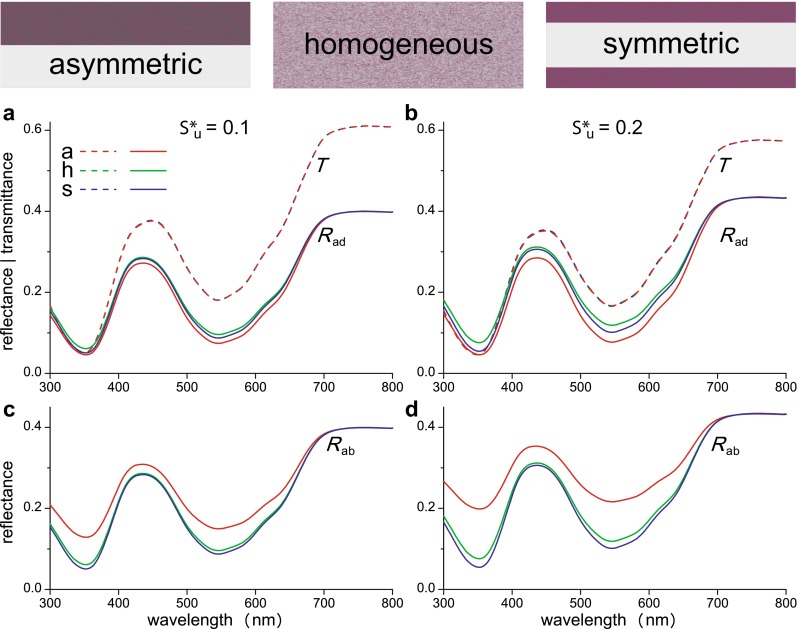
Transmittance and reflectance spectra of a few differently organized model flower petals. **a**, **b** Transmittance (*T*) and adaxial reflectance (*R*_ad_) spectra. **c**, **d** Abaxial reflectance (*R*_ab_) spectra. The spectra are calculated for two values of the scattering parameter of the unpigmented layer, $S_{\text{u}}^{*}$: 0.1 (**a**, **c**) and 0.2 (**b**, **d**). The three model petals had the same amount of pigment and scattering distributed asymmetrically (*a*), homogenously (*h*), or symmetrically in the adaxial and abaxial layer (*s*)

**Fig. 6:**
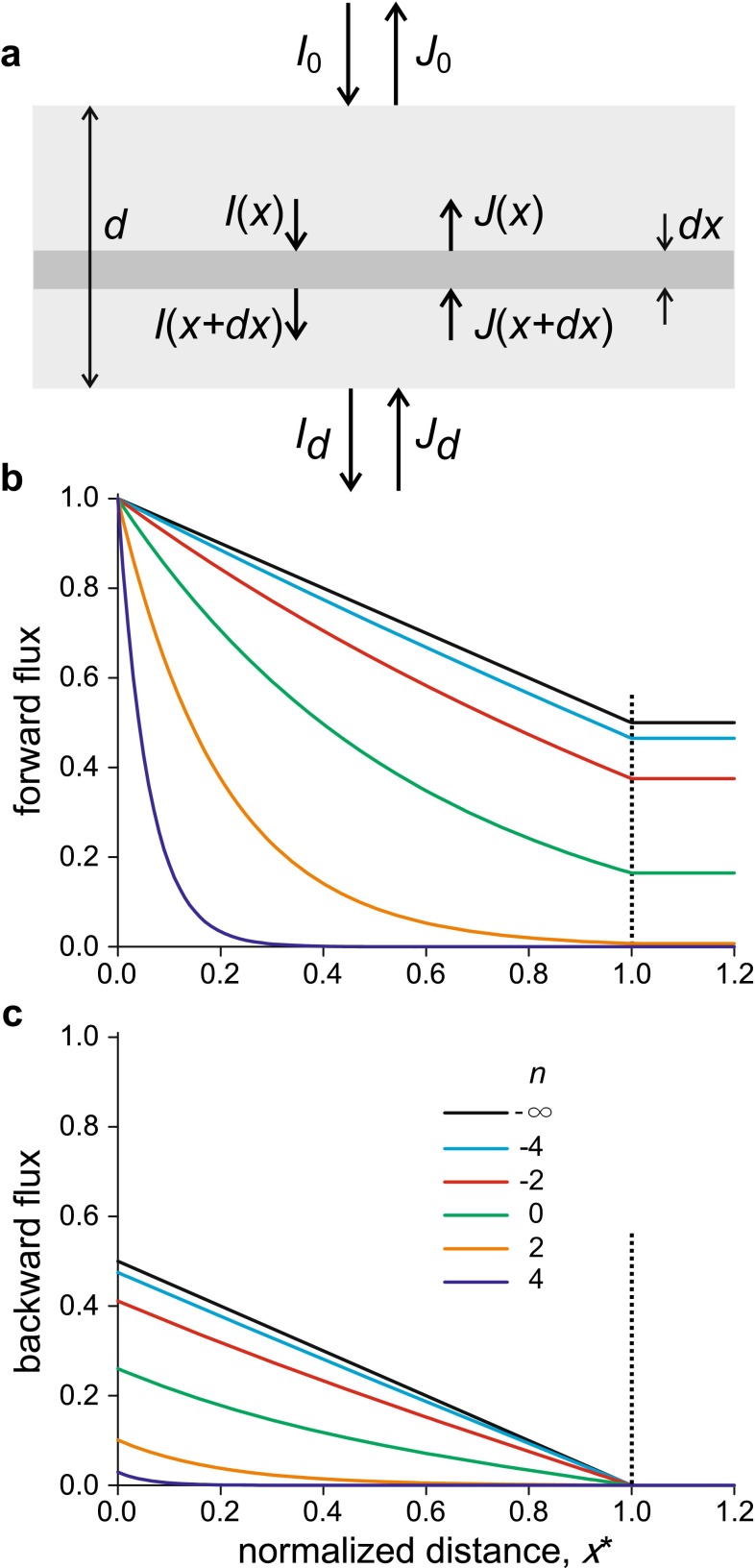
**a** Diagram for the light flux in a homogeneous, but scattering medium in a plate with thickness *d*, used in Kubelka–Munk theory. *I* is the light flux in the forward direction with *I*_0_ the incident light. *J* is the flux in the backward direction. **b** The forward light flux, *I*, in a homogeneous but absorbing and diffusively scattering medium, with thickness *d*, absorption coefficient *K* and scattering coefficient *S*, presented as a function of the normalized depth x* = *x/d*. In this example, a unit light flux enters the medium, the scattering parameter S* = *Sd* has the value S* = 1, and the ratio of the absorption coefficient and scattering coefficient is *r* = *K/S* = 2^*n*^, with *n* varying between −4 and +4; in the absence of absorption *n* = −∞: *K* = 0. **c** The backward light flux, *J*, associated with the forward light flux of (**b**)

To investigate the differences between various distributions of pigments quantitatively, we have calculated reflectance spectra for three flower cases (Fig. 5) a—asymmetric pigment distribution with two equally thick layers, one pigmented and one unpigmented (as in *N. paradoxa*); h—homogeneous pigment distribution throughout the petal, with the same total amount of pigment and scattering parameter; s—symmetric pigment distribution, where the same amount of pigment is equally deposited in two layers, adaxially and abaxially, with in between the purely scattering layer of the first case. We considered for all three cases the two previously encountered scattering parameters of the unpigmented layer, $S_{u}^{*}$ = 0.1 (Fig. 5a, c) and 0.2 (Fig. 5b, d).

For each of the three cases, the calculated adaxial and abaxial transmittance spectra are of course identical (Fig. 5a, b). The calculated adaxial (*R*_ad_) and abaxial (*R*_ab_) reflectance spectra are identical for both the homogeneous and symmetric case, and the spectral differences between the two cases are small. However, an asymmetric localization of pigment yields very different adaxial and abaxial reflectance spectra, and hence very different colours of both sides (Fig. 5c, d). Clearly, with pigment localized on only one side, the light reflected by that side is more saturated than when the pigment is distributed homogeneously or symmetrically (Fig. 5). This is because the asymmetrically located pigment is most efficiently used with illumination of the pigmented side: the light backscattered by the unpigmented layer traverses the pigmented layer twice. Pollinators are capable of detecting very small differences in floral spectra and are especially sensitive for the degree of saturation (Lunau 1990; Renoult et al. 2013; Rohde et al. 2013). Obviously, for flowers that will be visited by pollinators from only one side the most efficient strategy is an asymmetrical deposition of pigment.

In conclusion, the combined Kubelka–Munk-layer-stack model allows a heuristic approach to understand flower coloration. It enables a quantitative analysis of the relative importance of pigmentation and scattering. The approach presented here will help us to understand how plants can optimize their coloration for attracting pollinators as well as to explain the functional causes as to why plants use various flower coloration strategies.

### *Author contribution statement*

Both authors performed the experiments and wrote the paper. DGS applied the theory and performed the main calculations.

## Figures and Tables

**Fig. 7:**
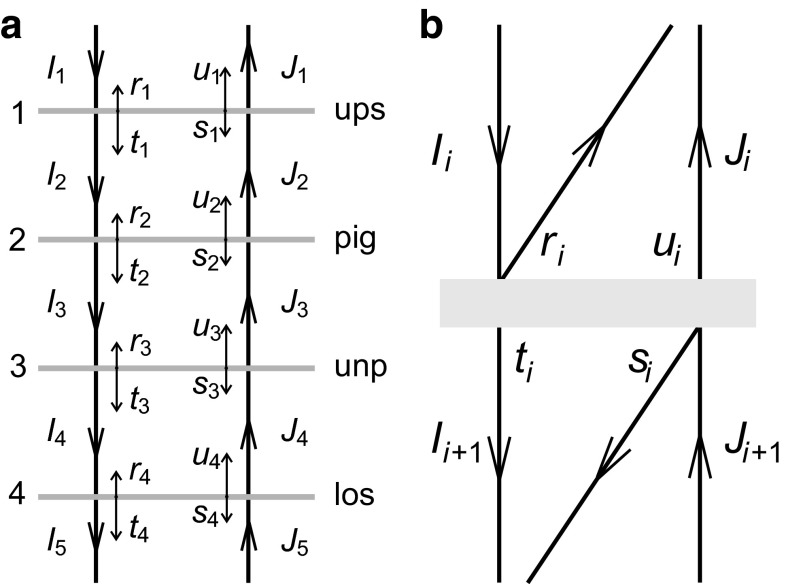
Light propagation in a stack of four layers as a model for the flower of *N. paradoxa*. **a** The model flower consists of an upper surface (*ups*), a pigment layer (pig), an unpigmented layer (*unp*), and a lower surface (*los*). **b** Each layer in **a** (with number *i* = 1 − 4) has characteristic reflectances *r*_*i*_ and *s*_*i*_ and transmittances *t*_*i*_ and *u*_*i*_, with forward incident and transmitted light fluxes *I*_*i*_ and *I*_*i*+1_ and backward incident and transmitted light fluxes *J*_*i*+1_ and *J*_*i*_. The* forward* and* backward* light fluxes between the layers are sums of transmitted and reflected components

## Appendix

### Kubelka–Munk theory of absorbing and scattering media

The light flux in a flower was modelled by using the Kubelka–Munk theory for absorbing and scattering media (Kubelka and Munk 1931), which assumes a homogeneous, pigmented slab with thickness *d*, and with absorption coefficient *K* and scattering coefficient *S* for randomly propagating light (Fig. 6a). Light entering the medium will be partly absorbed and scattered, resulting in a diffuse forward propagating light flux *I* and a backward propagating light flux *J*. The change in these light fluxes in an infinitesimal layer with thickness *dx* is described by:

1a $$I\left( {x + dx} \right) = I\left( x \right) - KI\left( x \right)dx - SI\left( x \right)dx + SJ\left( x \right)dx$$

1b $$J\left( x \right) = J\left( {x + dx} \right) - KJ\left( x \right)dx - SJ\left( x \right)dx + SI\left( x \right)dx$$

or,

2a $$dI/dx = - \left( {K + S} \right)I + SJ$$

2b $$dJ/dx = - SI + \left( {K + S} \right)J$$

The general solution of the two coupled linear differential equations is, assuming a unit incident light flux, *I*_0_ = 1 at *x* = 0, and a zero backward light flux *J*_*d*_ = 0 at *x* = *d*:

3a $$I\left( x \right) = \left\{ {\left( {a + b} \right)\exp \left[ {c\left( {d - x} \right)} \right] - \left( {a - b} \right)\exp \left[ { - c\left( {d - x} \right)} \right]} \right\}/N$$

3b $$J\left( x \right) = \left\{ {\exp \left[ {c\left( {d - x} \right)} \right] - \exp \left[ { - c\left( {d - x} \right)} \right]} \right\}/N$$

with

3c $$N = \left( {a + b} \right)\exp \left( {cd} \right) - \left( {a - b} \right)\exp \left( { - cd} \right)$$

and

3d $$a = 1 + K/S,\quad b = \sqrt {a^{2} - 1} ,\quad c = bS$$

Normalizing all parameters to the total thickness *d*, Eqs. 3a–3d yield

4a $$I(x^{*} ) = \{ A^{*} { \sinh }[B^{*} \left( {1 - x^{*} } \right)] + B^{*} { \cosh }B^{*} \left( {1 - x^{*} } \right)\} /\{ A^{*} { \sinh }B^{*} + B^{*} { \cosh }B^{*} \}.$$

4b $$J(x^{*} ) = S^{*} { \sinh }[B^{*} \left( {1 - x^{*} } \right)]/\{ A^{*} { \sinh }B^{*} + B^{*} { \cosh }B^{*} \}$$

where

4c $$S^{*} = Sd,\;A^{*} = aS^{*} ,\;B^{*} = bS^{*} = cd,\;x^{*} \, = x/d .$$

As an example, Fig. 6b, c present the forward and backward light fluxes, calculated with Eqs. 4a–4c, as a function of the normalized distance *x** for a number of values of the ratio of the absorption coefficient and scattering coefficient, *r* = *K/S* = 2^*n*^, with *n* varying between −4 and +4, assuming a scattering parameter *S** = 1. It follows from Eqs. 4a–4c that the reflectance *R* = *J*_0_ and the transmittance *T* = *I*_d_ are:

5a $$R = (S^{*} { \sinh }B^{*} )/(A^{*} { \sinh }B^{*} + B^{*} { \cosh }B^{*} )$$

5b $$T = B^{*} /(A^{*} { \sinh }B^{*} + B^{*} { \cosh }B^{*} ).$$

From Eqs. 5a, 5b the scattering and absorption parameters can be derived (Yamada and Fujimura 1991):

6a $$S^{*} = [\ln \{ (1 - \left( {a - b} \right)R)/T\} ]/b.$$

and

6b $$K^{*} = \left( {a - 1} \right)S^{*}$$

with

6c $$a = (1 + R^{2} - T^{2} )/(2R),\quad b = \sqrt {a^{2} - 1}.$$

Therefore, when the thickness *d* is known, the scattering and absorption coefficients, *S* and *K*, can be obtained by measuring the reflectance and transmittance spectra.

The general Kubelka–Munk case has two extreme limits, namely no scattering (*S* = 0) and no absorption (*K* = 0). When *S* = 0, there is only a forward light flux, or *J*(*x*) = 0 throughout the medium. Then Eq. 2a yields $I\left( x \right) = { \exp }( - Kx)$, the classical Lambert–Beer law. The absorbance (or optical density) of a layer with thickness *d* then is *D* = −log_10_(${ \exp }( - Kd)$) = 0.4343*Kd* = 0.4343*K**, with *Kd* = *K**.

When *K* = 0, Eqs. 2a and 2b yield

7 $$dI/dx = dJ/dx = S\left( {J - I} \right) = {\text{const}} .$$

Hence, with *I*_0_ = 1 and *J*_*d*_ = 0, it follows that.

8a $$I\left( x \right) = Sx/(1 + Sd)$$

8b $$J\left( x \right) = S(d - x)/(1 + Sd)$$

or, with Eq. 4c:

9a $$I\left( {x^{*} } \right) = 1 - S^{*} x^{*} /(1 + S^{*} )$$

9b $$J\left( {x^{*} } \right) = S^{*} (1 - x^{*} )/ (1 + S^{*} )$$

The reflectance and transmittance of the slab then are

10a $$R = S^{*} /(1 + S^{*} )$$

10b $$T = { 1}/( 1+ S^{ * } ).$$

For a non-absorbing medium, the scattering parameter thus follows directly from its reflectance

11 $$S^{*} = R/(1 - R) .$$

and, when the thickness *d* is known, the scattering coefficient *S* = *S*/d* is immediately obtained.

### Reflectance and transmittance of a stack of layers

We consider a stack of *n* layers, where each layer *i* has a characteristic reflectance *r*_*i*_ and transmittance *t*_*i*_ for forward propagating light *I*_*i*_, and *s*_*i*_ and *u*_*i*_ for backward propagating light *J*_*i*_ (Fig. 7a, b). Hence

12a $$I_{i + 1} = t_{i} I_{i} + s_{i} J_{i + 1}.$$

and

12b $$J_{i} = u_{i} J_{i + 1} + r_{i} I_{i} .$$

Equations 12a, 12b are equivalent to (see Yamada and Fujimura 1991)

13 $$\left( {\begin{array}{*{20}c} {I_{i + 1} } \\ {J_{i + 1} } \\ \end{array} } \right) = M_{i} \left( {\begin{array}{*{20}c} {I_{i} } \\ {J_{i} } \\ \end{array} } \right)\;\quad{\text{with}}\;M_{i} = \frac{1}{{u_{i} }}\left[ {\begin{array}{*{20}c} {u_{i} t_{i} - r_{i} su_{i} } & {s_{i} } \\ { - r_{i} } & 1 \\ \end{array} } \right] .$$

Although in general the reflectance values *r*_*i*_ and *s*_*i*_ as well as transmittance values *t*_*i*_ and *u*_*i*_ may differ, we may assume that layers can be chosen so that *s*_*i*_ = *r*_*i*_ and *u*_*i*_ = *t*_*i*_. The layer transfer matrix then is

14 $$M_{i} = \frac{1}{{t_{i} }}\left[ {\begin{array}{*{20}c} {t_{i}^{2} - r_{i}^{2} } & {r_{i} } \\ { - r_{i} } & 1 \\ \end{array} } \right]$$

For a stack of *n* layers Eq. 13 yields

15 $$\left( {\begin{array}{*{20}c} {I_{n + 1} } \\ {J_{n + 1} } \\ \end{array} } \right) = M_{\text{s}} \left( {\begin{array}{*{20}c} {I_{1} } \\ {J_{1} } \\ \end{array} } \right)\;{\text{with}}\;M_{\text{s}} = M_{n} M_{n - 1} \ldots M_{1} = \mathop \prod \limits_{i = 1}^{n} M_{n + 1 - i} = \left[ {\begin{array}{*{20}c} {m_{11} } & {m_{12} } \\ {m_{21} } & {m_{22} } \\ \end{array} } \right].$$

For illumination from above (the side of plate 1 in Fig. 7a), the stack transmittance is $T_{\text{a}} = I_{n + 1} /I_{1}$ and the stack reflectance is $R_{\text{a}} = J_{1} /I_{1}$. With $J_{n + 1} = 0$ and det(*M*_s_) = det(*M*_*i*_) = 1 it then follows that $T_{\text{a}} = 1/m_{22}$ and $R_{\text{a}} = - m_{21} /m_{22}$. For illumination from below, i.e., the side of plate *n*, Eq. 15 also holds, because of reversibility of light rays, but now the stack transmittance is $T_{\text{b}} = J_{1} /J_{n + 1}$ and the stack reflectance is $R_{\text{b}} = I_{n + 1} /J_{n + 1}$with $I_{1} = 0$ it then follows that $T_{\text{b}} = 1/m_{22}$ and $R_{\text{b}} = m_{12} /m_{22}.$ In other words, the transmittances are the same for illumination from either side of a stack, *T* = *T*_a_ = *T*_b_, but the reflectances may be different.

The stack transfer matrix $M_{\text{s}},$ defined by

16 $$\left( {\begin{array}{*{20}c} T \\ 0 \\ \end{array} } \right) = M_{\text{s}} \left( {\begin{array}{*{20}c} 1 \\ {R_{\text{a}} } \\ \end{array} } \right)\;\quad{\text{or}}\;\left( {\begin{array}{*{20}c} {R_{\text{b}} } \\ 1 \\ \end{array} } \right) = M_{\text{s}} \left( {\begin{array}{*{20}c} 0 \\ T \\ \end{array} } \right).$$

can be determined by measuring the transmittances and reflectances, because

17 $$M_{\text{s}} = \frac{1}{T}\left[ {\begin{array}{*{20}c} {T^{2} - R_{\text{a}} R_{\text{b}} } & {R_{\text{b}} } \\ { - R_{\text{a}} } & 1 \\ \end{array} } \right].$$

We note that this matrix has the same formal shape as that for a single layer (Eq. 14) when the reflectances for illumination from above and below are equal, *R*_a_ = *R*_b_.

Finally, when the optical properties of a single layer with number *j* are unknown, its transfer matrix *M*_*j*_ can be derived from

18 $$M_{j} = \mathop \prod \limits_{i = j + 1}^{n} M_{i}^{ - 1} M_{\text{s}} \mathop \prod \limits_{i = 1}^{j - 1} M_{i}^{ - 1} .$$

so that the transmittance and reflectance of layer *j* then can be obtained with *i* = *j* from Eq. 14.

As an alternative to the above formal analysis, a slightly more intuitive approach is outlined below. Equations 12a, 12b yield that the transmittance parameter of each layer is

19a $$\tau_{i} = I_{i + 1} /I_{i} = t_{i} /(1 - s_{i} \rho_{i + 1} ).$$

and the reflectance parameter is

19b $$\rho_{i} = J_{i} / I_{i} = r_{i} + u_{i} \rho_{i + 1} \tau_{i}$$

With *s*_*i*_ = *r*_*i*_ and *u*_*i*_ = *t*_*i*_

20a $$\rho_{i} = r_{i} + t_{i} \rho_{i + 1} \tau_{i}$$

and

20b $$\tau_{i} = t_{i} /(1 - r_{i} \rho_{i + 1} ) .$$

from Eqs. 20a, 20b it follows that

21a $$\rho_{i} = r_{i} + t_{i}^{2} \rho_{i + 1} /(1 - r_{i} \rho_{i + 1} )$$

and equivalently that

21b $$\rho_{i + 1} = (\rho_{i} - r_{i} )/ [t_{i}^{2} + r_{i} (\rho_{i} - r_{i} ) ].$$

With light incident only from one side, *J*_*n*+1_ = 0, or, *ρ*_*n*+1_ = 0, so that, when the reflectance and transmittance values for all layers are known, the stack reflectance *R*_s_ = *J*_1_/*I*_1_ = *ρ*_1_ is obtained by subsequently calculating *ρ*_*i*_ for *i* = *n*, *n* − 1,.., 2, 1. The transmittance *T*_s_$= I_{n + 1} /I_{1} = \mathop \prod \nolimits_{1}^{n} \tau_{i}$ follows from Eq. 20b by subsequently calculating $\tau_{i}$ for *i* = 1, 2,.., *n*.

For the special case of *N. paradoxa*, layers 1 and 4 are the surfaces of the adaxial and abaxial epidermis. Layers 2 and 3 are inhomogeneous and thus will scatter propagating light. Layer 3 is assumed to be unpigmented, and therefore the reflectance and transmittance values are determined by only the scattering coefficient, which will be about constant, that is, independent of the light wavelength. The reflectance and transmittance of layer 3 hence are described by Eqs. 10a, 10b. However, in the pigmented layer 2 the absorption and scattering coefficient, and thus its reflectance and transmittance, will be wavelength dependent.

We can estimate the reflectance and transmittance of layer 2 with the procedure outlined below. We therefore consider again a stack of *n* layers, but now the reflectance and transmittance of all layers are known except one, that with number *j*. Then, with illumination from the side of layer 1, by measuring the stack reflectance *R*_s_ = *ρ*_1_, we can calculate *ρ*_*i*_ with *i* = 2, 3…*j* by using Eq. 21b with *i* = 1, 2,…*j* *−* 1. The other reflectance parameters *ρ*_*i*_ can be calculated by using Eq. 21a and *ρ*_*n*+1_ = 0, going from *i* = *n*, *n *− 1, …to *i* = *j* + 1. Subsequently, with Eq. 20b we can calculate *τ*_*i*_ for *i* = 1…*j* − 1 and *i* = *j* + 1…*n*. By measuring the stack transmittance *T*_s_, we then can calculate $\tau_{j} = T_{\text{s}} /(\mathop \prod \nolimits_{1}^{j-1} \tau_{i} \mathop \prod \nolimits_{j + 1}^{n} \tau_{i} ).$ Having thus obtained the compound reflectance and transmittance parameters *ρ*_*j*_ and *τ*_*j*_, we can derive the reflectance and transmittance of layer *j*, *r*_*j*_ and *t*_*j*_, because Eqs. 20a, 20b are equivalent to

16a $$t_{j} = \tau_{j} (1 - \rho_{j} \rho_{j + 1} )/[1 - \rho_{j + 1}^{2} \tau_{j}^{2} ].$$

and

16b $$r_{j} = \rho_{j} - t_{j} \rho_{j + 1} \tau_{j} .$$

Finally, with *r*_*j*_ = *R* and *t*_*j*_ = *T* we derive from Eqs. 6a–6c the scattering and absorption parameters of layer *j*, *S** and *K**, and with the thickness *d* of layer *j*, the scattering and absorption coefficients *S* = *S*/d* and *K* = *K*/d* are obtained.

## References

[CR1] Allen WA, Gausman HW, Richardson AJ (1969). Interaction of isotropic light with a compact plant leaf. J Opt Soc Am.

[CR2] Allen WA, Gausman HW, Richardson AJ (1969). Mean effective optical constants of cotton leaves. J Opt Soc Am.

[CR3] Arnold SE, Faruq S, Savolainen V, McOwan PW, Chittka L (2010). FReD: the floral reflectance database—a web portal for analyses of flower colour. PLoS One.

[CR4] Chittka L, Menzel R (1992). The evolutionary adaptation of flower colours and the insect pollinators’ colour vision. J Comp Phys A.

[CR5] Dyer AG, Boyd-Gerny S, McLoughlin S, Rosa MG, Simonov V, Wong BB (2012). Parallel evolution of angiosperm colour signals: common evolutionary pressures linked to hymenopteran vision. Proc R Soc B.

[CR6] Exner F, Exner S (1910). Die physikalischen Grundlagen der Blütenfärbungen. Sitzungsber Kais Akad Wiss Wien Math-Nat Kl I.

[CR7] Gausman HW, Allen WA, Escobar DE (1974). Refractive index of plant cell walls. Appl Opt.

[CR8] Gkikas D, Argiropoulos A, Rhizopoulou S (2015). Epidermal focusing of light and modelling of reflectance in floral-petals with conically shaped epidermal cells. Flora.

[CR9] Gorton HL, Vogelmann TC (1996). Effects of epidermal cell shape and pigmentation on optical properties of *Antirrhinum* petals at visible and ultraviolet wavelengths. Plant Physiol.

[CR10] Grotewold E (2006). The genetics and biochemistry of floral pigments. Annu Rev Plant Biol.

[CR11] Kay Q, Daoud H, Stirton C (1981). Pigment distribution, light reflection and cell structure in petals. Bot J Linn Soc.

[CR12] Kubelka P, Munk F (1931). Ein Beitrag zur Optik der Farbanstriche. Z Techn Physik.

[CR13] Lee DW (2007). Nature’s palette: the science of plant color.

[CR14] Lee DW (2009) Plant tissue optics: micro- and nanostructures. Proc. SPIE 7401, Biomimetics and Bioinspiration, 740104

[CR15] Lunau K (1990). Colour saturation triggers innate reactions to flower signals: flower dummy experiments with bumblebees. J Comp Physiol A.

[CR16] Papiorek S, Junker RR, Lunau K (2014). Gloss, colour and grip: multifunctional epidermal cell shapes in bee-and bird-pollinated flowers. PLoS One.

[CR17] Renoult JP, Thomann M, Schaefer HM, Cheptou PO (2013). Selection on quantitative colour variation in Centaurea cyanus: the role of the pollinator’s visual system. J Evol Biol.

[CR18] Rohde K, Papiorek S, Lunau K (2013). Bumblebees (*Bombus terrestris*) and honeybees (*Apis mellifera*) prefer similar colours of higher spectral purity over trained colours. J Comp Physiol A.

[CR19] Stavenga DG, Giraldo MA, Hoenders BJ (2006). Reflectance and transmittance of light scattering scales stacked on the wings of pierid butterflies. Opt Express.

[CR20] van der Kooi CJ, Wilts BD, Leertouwer HL, Staal M, Elzenga JTM, Stavenga DG (2014). Iridescent flowers? Contribution of surface structures to optical signaling. New Phytol.

[CR21] van der Kooi CJ, Dyer AG, Stavenga DG (2015). Is floral iridescence a biologically relevant cue in plant-pollinator signaling?. New Phytol.

[CR22] van der Kooi CJ, Pen I, Staal M, Stavenga DG, Elzenga JTM (2015). Competition for pollinators and intracommunal spectral dissimilarity of flowers. Plant Biol.

[CR23] Wehner R, Bernard GD (1993). Photoreceptor twist: a solution to the false-color problem. Proc Natl Acad Sci USA.

[CR24] Yamada N, Fujimura S (1991). Nondestructive measurement of chlorophyll pigment content in plant leaves from three-color reflectance and transmittance. Appl Opt.

[CR25] Zelko I, Lux A, Sterckeman T, Martinka M, Kollárová K, Lišková D (2012). An easy method for cutting and fluorescent staining of thin roots. Ann Bot.

